# Mass balance, pharmacokinetics, metabolism, and excretion of radiolabeled acoziborole, a potential novel treatment for human African trypanosomiasis, following single microtracer oral dose to humans

**DOI:** 10.1128/aac.00580-25

**Published:** 2025-09-22

**Authors:** Jean-Yves Gillon, François Simon, Sharan Sidhu, Mathieu Louis, Delphine Launay, Valérie Wauthier, Marta Pelay-Gimeno, Lotte van Andel, Sabrina Loyau, Sandra Rembry, Estelle Weinling, Antoine Tarral

**Affiliations:** 1 Drugs for Neglected Diseases initiative (DNDi) 58076, Geneva, Switzerland; 2 Quotient Sciences 416201 , Nottingham, United Kingdom; 3 SGS Belgium S.A., Wavre, Belgium; 4 TNO, Leiden, the Netherlands; 5 PhinC Development, Massy, France; 6 Sanofi R&D, Pharmacokinetics, Dynamics and Metabolism, Montpellier, France; The Children's Hospital of Philadelphia, Philadelphia, Pennsylvania, USA

**Keywords:** antiparasitic, mass balance, phase I, human African trypanosomiasis

## Abstract

Acoziborole is an oxaborole 6-carboxamide active against *Trypanosoma brucei gambiense* and *rhodesiense*, the parasites responsible for human African trypanosomiasis. This open-label, phase I study in six healthy male participants evaluated the mass balance, pharmacokinetics, metabolism pathways, and excretion of a single oral 960 mg dose of [^14^C]-acoziborole. Blood, plasma, and feces were collected for 120 days and urine for 16 days. The excretion balance and systemic exposure of total circulating radioactivity were determined using accelerator mass spectrometry. Metabolism profiling was performed in pools of plasma, urine, and feces. Liquid chromatography coupled with tandem mass spectrometry quantified acoziborole and its metabolite SCYX-3109 in plasma and urine. By Day 60, 87.3% of the total radioactivity had been recovered (10.9% and 74.2% of the total dose in urine and in feces, respectively), with an excretion increment of <1% between Days 59 and 61. Mean ratios of total radioactivity indicated an equivalent distribution between blood cells and plasma. The concentration-time profiles of total radioactivity and acoziborole in plasma were similar. In plasma, acoziborole accounted for 95.1%, an oxidized form of acoziborole for 2.3% of the total radioactivity, while SCYX-3109 was not detected. Seven metabolites (oxidative deboronation, glucuronidation, and mono-oxidation) were detected in urine with individual abundances relative to the dose of 0.1–2.1%; unchanged acoziborole accounted for 0.6%. In feces, acoziborole, SCYX-3109, and two other oxidized or deboronated forms of acoziborole represented 33.6%, 12.3%, and 2.3% of the dose, respectively. Acoziborole showed good absorption, limited metabolism, minimal urinary elimination, and predominant but slow biliary-fecal elimination.

## INTRODUCTION

Acoziborole (SCYX-7158) is a first-in-class oxaborole 6-carboxamide derivative active against *Trypanosoma brucei (T.b.) gambiense* and *rhodesiense*, the parasites responsible for human African trypanosomiasis (HAT) (1, 2). HAT, also known as “sleeping sickness,” is a life-threatening condition transmitted by tsetse flies that is considered endemic in 36 countries, mainly in rural areas of sub-Saharan Africa. The disease occurs in two clinical stages: during the early hemolymphatic stage (stage 1), trypanosomes reside in the blood and lymphatic system, resulting in clinical signs and symptoms that are mild and non-specific. In the later meningoencephalitic stage (stage 2), the parasites invade the central nervous system and patients exhibit characteristic neurological manifestations, such as disorientation, increasing sleep disturbances, and, eventually, coma and death (3–5).

Acoziborole has been selected from a series of benzoxaborole 6-carboxamide derivatives that showed good *in vitro* potency and efficacy in preclinical stage 1 and stage 2 mouse models of HAT (1, 4). Acoziborole targets the cleavage and polyadenylation specificity factor 3 (CPSF3) in *T.b. gambiense*, an endonuclease involved in the control of polyadenylation and trans-splicing of pre-mRNA (6). In mouse models of both acute and chronic *T.b. gambiense* infection, oral administration of acoziborole at doses of 5–25 mg/kg/day was found to be curative and to significantly prolong survival (1). Preclinical pharmacokinetic (PK) studies have indicated that acoziborole is well absorbed (absolute bioavailability of 55% and 89%, in mice and non-human primates [NHPs], respectively), when taken orally (1). Additionally, acoziborole has been proven to cross the blood-brain barrier, with brain to plasma AUC_0–24h_ ratio of 44% in rats, similar to that in mice. In NHPs, concentrations of acoziborole in the CSF were approximately 5% of the plasma concentration at each time point (1). In all animal species investigated so far, acoziborole was slowly metabolized through oxidation, resulting in the formation of at least one inactive metabolite (SCYX-3109) after oxidative deboronation (2). In addition, after single oral administration of [^14^C]-acoziborole (10 mg/kg) in Sprague-Dawley rats, plasma samples were taken at pre-dose, 6 h, 24 h and 96 h post-dose and urine and feces at 6–24, 24–48, and 48–72 h time intervals. Five metabolites were detected by radio HPLC and LC-MS/MS in plasma, urine, and feces, including SCYX-3109, or resulting from mono-oxidation of acoziborole, mono-oxidation of SCYX-3109, di-oxidation of SCYX-3109, and glucuronidation of SCYX- 3109.

In a single-dose, first-in-human phase I study in healthy sub-Saharan African male participants, plasma concentrations of acoziborole increased rapidly up to 24 h and remained steady up to 96 h post-dose, then decreased very slowly with a *t*_1/2_ of between 267 and 411 h (7). Moreover, acoziborole was widely distributed throughout the body, and unbound concentrations of 0.3–4.6% were found in cerebrospinal fluid, in the same range to that observed in mouse preclinical studies (0.3–3.9%) (7). In contrast, plasma concentrations of SCYX-3109 in humans were very low at all time points.

A clinical phase II/III, multicenter, open-label, prospective study in the Democratic Republic of the Congo and Guinea demonstrated an acceptable safety and tolerability profile in 208 male and female sub-Saharan African patients with confirmed *gambiense* HAT infection who were exposed to a single oral dose of 960 mg acoziborole in fasting conditions. The treatment success rate at 18 months was 95.2% in late-stage patients (modified intention-to-treat population) and 98.1% in 159 out of 162 patients (evaluable population) (8).

Given the importance of understanding the metabolism and elimination of acoziborole in humans, the objectives of this study were to characterize the mass balance, PK, metabolism, and excretion of the same single oral dose (960 mg) of [^14^C]-acoziborole in healthy participants.

## MATERIALS AND METHODS

### Study design and interventions

This single-center, open-label, single group, non-randomized, single oral dose study was performed at Quotient Sciences Ltd (Ruddington, Nottingham, UK). Clinical trial authorization was obtained from the Medicines and Healthcare Products Regulatory Agency (UK). Approval for administration of [^14^C]-acoziborole was obtained from an expert committee sponsored by the Department of Health and Social Care in the United Kingdom (Administration of Radioactive Substances Advisory Committee [ARSAC]), and from the ARSAC-accredited Quotient Sciences’ nuclear medicine practitioner. This study was approved by a local research ethics committee in the United Kingdom and was conducted in compliance with the Declaration of Helsinki and the International Conference on Harmonisation E6 Guideline for Good Clinical Practice. The study was registered in clinicaltrials.gov (NCT04270981) and in EudraCT (reference 2019-004059-35). Written informed consent was obtained from all participants before undertaking any study-related procedures.

Participants were admitted in the evening of the day before dosing (Day −1) and were dosed in the morning of Day 1, following an overnight fast from food and drink of a minimum of 9 h. They remained fasted until approximately 4 h post-dose, at which time lunch was provided. Participants remained resident in the clinic until up to 240 h after dosing (up to Day 11). Given the long acorziborole terminal half-life, participants were to return to the clinical unit for an additional 48-h collection period on Days 14–17, being admitted the evening before the collection period and for four subsequent outpatient visits on Days 31, 59, 89, and 119. However, due to the COVID-19 pandemic, instead of returning for the outpatient visits, participants were requested to perform home collection of feces for a 48-h period (i.e., Days 31–33, Days 59–61, Days 89–91, and Days 119–121). Collected samples were couriered to the clinical unit at the end of each collection period. A follow-up call took place 5–10 days after the final outpatient visit to ensure the well-being of participants.

### Study participants

Participants were healthy Caucasian males, aged between 18 and 55 years, with a body mass index (BMI) of 18–30 kg/m^2^. Participants were deemed in good health based on screening results of physical examinations, vital signs, 12-lead electrocardiograms (ECGs), clinical laboratory tests, and urine drug tests performed within 28 days prior to drug administration. Participants were not to have a history of drug hypersensitivity or allergic reactions related to any drug or formulation excipients. Other exclusion criteria included participants who had received any investigational medicinal product (IP) in a clinical research study within 90 days prior to Day 1; who were study site employees, or immediate family members; who had a history of any drug or alcohol abuse in the past 2 years; who had regular alcohol consumption of >21 units/week; who were current smokers or had smoked within the last 6 months; who had pregnant or lactating partners; or who had been exposed to radiation exceeding 5 mSv in the last 12 months or 10 mSv in the last 5 years. A full list of inclusion and exclusion criteria is available in Supplementary Information section 1.

### Radiolabeled acoziborole and dosage form

**Fig 1:**
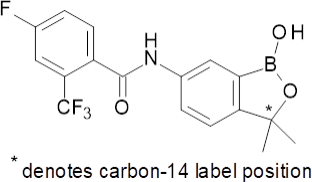
Chemical structure of [^14^C]-acoziborole (SCYX-7158).

Non-radiolabeled acoziborole was synthesized according to Good Manufacturing Practice (GMP) by Avista Pharma (now Cambrex, Durham NC, USA). [^14^C]-acoziborole was synthesized by Aptuit (Kansas City, USA); ^14^C was included in place of the dimethyl-substituted ^12^C atom (Fig. 1) with a specific activity of 0.157 mCi/mg. [^14^C]-acoziborole was mixed with non-radiolabeled acoziborole and further purified under GMP by Selcia Ltd (Ongar, UK) to a radiochemical purity of 99.3% and specific activity of 0.94 nCi/mg. Non-radiolabeled acoziborole was used to dilute [^14^C]-acoziborole to the target radioactivity level. [^14^C]-Acoziborole for oral administration was supplied as 240 mg hydroxypropyl methylcellulose capsules, containing not more than (NMT) 9.25 kBq (250 nCi) ^14^C, which were manufactured at Quotient Sciences Ltd (Nottingham, UK). Manufacturing, packaging, quality control, and preparation of clinical supplies complied with GMP guidelines. Subjects received an oral dose of 960 mg [^14^C]-acoziborole on a single occasion (four capsules containing a total of NMT 1,000 nCi [37 kBq] ^14^C) in fasting condition. The dose of radioactivity was determined following review of human dosimetry calculations provided by Public Health England, UK, based on data from the distribution study of [^14^C]-acoziborole in Sprague-Dawley rats. In this study, after a 10 mg/kg dose (200 µCi/kg), radioactivity collected in feces during 7 days represented over 66% and 73% of total radioactivity in males and females, respectively, and over 85% and 86% in combined feces and urine of males and females, respectively. The associated radiation exposure fell within International Commission on Radiological Protection guidelines (1992) for category I studies (≤0.1 mSv) (9, 10).

### Safety and tolerability assessment

Safety was assessed by adverse event (AE) reports and at specific times during the physical examination, vital signs, 12-lead ECGs, and laboratory evaluations (see Supplementary Information section 2). AEs were all recorded from the time participants signed the consent form until the follow-up call, 5–10 days after final study discharge, and were evaluated for type, severity, frequency of AEs, and relationship to the IP.

### Sample collection

Venous blood samples were collected via an indwelling cannula or by venepuncture. A 12 mL sample was taken into heparinized glass tube and chilled on ice immediately before IP administration and 3 mL samples were taken at 1, 4, 9, 12, 24, 48, 72, 96, 120, 144, 168, and 240 h and on Days 15, 31, 59, 89, and 119 after IP administration. The first 0.5 mL of blood withdrawn via cannula was discarded. One aliquot of heparinized whole blood (1 mL) per time point was stored at or below −20°C for total radioactivity analysis. The remaining blood was centrifuged, and the harvested plasma was stored at approximately −20°C for bioanalysis or transferred (exactly 1 mL) into polypropylene tubes pre-filled with 5 µL formic acid. One plasma sample (0.5 mL) was used for metabolite profiling while another (0.5 mL) was used for parent drug and SCYX-3109 metabolite analysis. The remaining plasma was used for total radioactivity analysis.

Urine samples were collected before IP administration (single urine void) and then during intervals of 0–6, 6–12, 12–24, 24–48, 48–72, 72–96, 96–120, 120–168, 168–192, 192–216, and 216–240 h post-dose and for the additional 48-h collection period starting on Day 15 post-dose, that is, 336–384 h. Samples were acidified with citric acid to avoid acoziborole degradation and were refrigerated until the end of the block collection periods. The total weight and volume of urine were then recorded. At the end of each collection period, two aliquots of acidified urine (20 mL) were taken from the sample and stored in polypropylene tubes at −20°C until further analysis for total radioactivity. Two aliquots of 1 mL acidified urine were collected into tubes containing bovine serum albumin in aqueous solution (to avoid non-specific adsorption of acoziborole) and NaCl for acoziborole and SCYX-3109 assays. Urine samples for metabolite profiling were pooled for each participant according to the time intervals reported above, from 0 to 240 h.

Feces were collected before dosing and at block intervals of 0–6, 6–12, 12–24, 24–48, 48–72, 72–96, 96–120, 120–168, and 168–240 h post-dose and for five additional 48-h home collection periods starting on Days 15, 29, 59, 89, and 119. At the end of each collection period, a portion of the sample (50 g) was retained and stored at approximately −20°C until analyzed. The remaining fecal homogenate was combined with that collected at the end of each time interval and stored at approximately −20°C.

### Plasma and urine assays

SGS Belgium (Wavre, Belgium) performed the analysis to determine acoziborole and SCYX-3109 concentrations, using validated bioanalytical assays with liquid chromatography with tandem mass spectrometry (LC-MS/MS) with a Supelco Ascentis Express C8, 2.7 µm, 50 × 2.1 mm I.D. separation column. In plasma, the lower limit of quantification (LLOQ) was 25 ng/mL for acoziborole and 10 ng/mL for SCYX-3109. In urine, the LLOQ was 10 ng/mL for both acoziborole and SCYX-3109.

### Determination of total radioactivity

TNO (Leiden, the Netherlands) performed the analysis of total radioactivity and the metabolite profiling and identification. All blood, plasma, urine, and fecal homogenate samples were analyzed for total radioactivity by accelerator mass spectrometry (AMS). AMS analysis was performed on a 1 MV multi-element AMS, model 4110 Bo, high voltage engineering (software: AMS 155), using Australian National University (ANU) sucrose-8542 (C_12_H_22_O_11_) with a certified ^14^C/^12^C isotope ratio (10) as the system suitability test sample and [^14^C]-paracetamol as an AMS quality check sample.

For each (diluted) sample, 5 µL blood, 5 µL plasma, 15 µL urine, or 30 mg fecal homogenate prepared in 1% CMC solution (1% CMC solution:feces ratio of 4:1) were transferred to tin foil cups. The samples were dried under a stream of nitrogen and subsequently placed in the elemental analyzer (EA, Vario Micro, Elementar, Germany), which acts as an autosampler and combustion device for the AMS. Single samples of blood, plasma, and urine were analyzed. Fecal homogenates were analyzed in triplicate, and the averaged value was reported. Total radioactivity in plasma and blood was expressed as ng equivalents (Eq) of acoziborole/mL. The LLOQ was 47.3 ngEq/mL in plasma (1.51 mBq/mL), 165.4 ngEq/mL in blood (5.28 mBq/mL), 33.8 ngEq/mL in urine (1.08 mBq/mL), and 87.1 ngEq/mL in feces (2.78 mBq/mL).

### Metabolite profiling and identification

Individual pools of plasma (0–336 h, including time points at 1, 4, 9, 12, 24, 48, 72, 96, 120, 168, 240, and 336 h), urine (0–240 h, including time intervals 0–6, 6–12, 12–24, 24–48, 48–72, 72–96, 96–120, 120–168, 168–192, 192–216, and 216–240 h) and feces homogenates (0–240 h, including time intervals 0–6, 6–12, 12–24, 24–48, 48–72, 72–96, 96–120, 120–168, and 168–240 h) were prepared; for plasma this was done according to the method described in reference 11 and for feces by using equal percentages per time point based on total volume. Subsequently, a pool per matrix was prepared by mixing equal volumes of each participant pool. In addition, a plasma pre-dose pool was prepared by mixing equal volumes of each pre-dose sample. For urine and feces, TNO blank pool was used to generate a pre-dose sample. Samples were stored below −18°C. Samples (100 µL plasma, 250 µL urine, and 200 mg feces) were pre-treated and injected onto an ultra-high performance liquid chromatography (UPLC)-MS system (Acquity UPLC system [Waters] coupled to a Q-Exactive [Thermo]; Acquity HSS C18 VanGuard 5 mm × 2.1 mm, 1.8 µm [Waters] pre-column and Acquity HSS C18 100 mm × 2.1 mm, 1.8 µm d.p. [Waters]). Fractions were collected and analyzed off-line by AMS. AMS analysis was performed on an AMS system, consisting of an elemental analyzer, a double gas interface, and a 1 MV multi-element AMS. All fecal homogenate sample analyzes were performed in triplicate. Plasma pre-dose and TNO blank urine and feces pools were used for background correction. The UPLC method qualification included determination of retention time stability and extraction recovery. Metabolites were tentatively assigned to fractions in which activity greater than the baseline was observed, based on retention time and structural information provided by DNDi. Presence of the metabolite was confirmed by accurate mass data from high-resolution mass spectrometric analysis.

### PKs and statistical analyses

PK parameters were calculated based on actual sampling times using standard non-compartmental analysis and WinNonlin version 8.1 software (Certara, Princeton, NJ, USA).

For total radioactivity in plasma and whole blood, *C*_max_, *t*_max_, *t*_1/2_, AUC_0–240h_, AUC_0–t_ and AUC_0–∞_ were reported. Blood-to-plasma ratio was calculated based on *C*_max_ and AUC values. For total radioactivity excreted in urine, feces, and both combined, the following parameters were calculated: total amount of total radioactivity excreted over the time interval between *t*_1_ and *t*_2_ (Ae_*t*1*−t*2_), Ae_*t*1−*t*2_ expressed as a percentage of the radioactive dose administered (%Ae_*t*1−*t*2_), CumAe, and Cum%Ae. For CumAe calculation, the recovery of total radioactivity during WinNonlin time intervals in which urine and feces were not collected was estimated using the area under the excretion rate-time curve from the end of the preceding collection interval to the start of the subsequent collection interval.

For acoziborole and SCYX-3109 in plasma, maximum concentration (*C*_max_), time to reach *C*_max_ (*t*_max_), elimination half-life (*t*_1/2_), area under the plasma concentration-time curve (AUC) from time 0 (pre-dose) to 240 h post-dose (AUC_0–240h_), AUC from time 0 to the time of the last measurable concentration (AUC_0–*t*_) and AUC from time 0 to infinity (AUC_0–∞_) were reported, as well as the apparent total body clearance (CL/F) and apparent volume of distribution during the terminal phase (*V*_z_/*F*) for acoziborole. The SCYX-3109 to parent ratios were calculated based on *C*_max_ and AUC_0–∞_.

In urine, the following parameters were reported: the cumulative amount of acoziborole and SCYX-3109 excreted (CumAe) in urine up to 240 h, 384 h, and time of last measurable concentration post-dose (CumAe_0–240h_, CumAe,_0–384h_, and CumAe_0–*t*_, respectively), the CumAe in urine expressed as a percentage of the dose administered up to 240 h, 384 h, and time of last measurable concentration post-dose (Cum%Ae_0–240h_, Cum%Ae_0–384h_, and Cum%Ae_0–*t*_, respectively) and renal clearance (CLr, calculated as CumAe_0–240h_/AUC_0–240h_).

Descriptive statistics are presented as numbers of observations (*N*), arithmetic means, medians, standard deviations (SD), and coefficients of variation (CV%).

## RESULTS

### Participants

Six healthy Caucasian male participants aged 26–55 (mean 36.3) years, with BMIs of 24.5–28.8 (mean 26.5) kg/m^2^ participated in the study (Table 1). All participants who received the IP and had biological samples collected for up to 240 h after drug administration were considered evaluable and were included in the safety and PK analyses (*N* = 6). All participants except one had blood, plasma, and feces samples collected for up to 2,880 h.

**TABLE 1:** Demographic characteristics of participants^*a*^

Characteristics	Total *N* = 6
Sex	
Male, n (%)	6 (100)
Age (years)	
Mean (SD)	36.3 (13.1)
Median (range)	29.5 (26–55)
Race, n (%)	
Caucasian	6 (100)
Weight (kg)	
Mean (SD)	80.5 (5.9)
Median (range)	80.6 (72.0–88.2)
BMI	
Mean (SD)	26.5 (1.5)
Median (range)	26.6 (24.5–28.8)
Alcohol use	
n (%)	3 (50)

^*a*^ BMI, body mass index; *n*, number of participants; SD, standard deviation.

### Study performance

PK, mass balance, and metabolite profiling and identification samples were obtained until Day 15 (360 h post-dose). During the clinical conduct of the study, the emergence of the global COVID-19 pandemic and the accompanying restrictions on travel and movement necessitated changes to the protocol. Admission to the clinical units for residency did not occur after Day 15. Blood and plasma samples were collected up to 240 h post-dose as per the original schedule of assessments. The amendment changed the return visits from 48-h residencies in the clinical unit to short ambulatory visits followed by home collection periods for fecal samples. Collection of urine samples was not possible in the absence of the additional residency periods given the complexity of preparing urine samples outside the investigational site.

### Safety and tolerability

Acoziborole was well-tolerated overall, and all reported treatment-emergent AEs (TEAEs, 13 in total in the six participants, see Supplementary Information section 3) were mild or moderate in severity and rapidly resolved. Most TEAEs (10/13) were not considered related to the IP. There were no significant changes in ECG parameters, vital signs, or physical examination post-dose. All mean laboratory testing (blood chemistry, hematology, and urinalysis) results were within acceptable range or considered non-clinically significant. There were no deaths, serious AEs, or participant discontinuations due to any AE reported in this study.

### PKs of radioactivity, acoziborole, and its metabolite SCYX-3109

**Fig 2:**
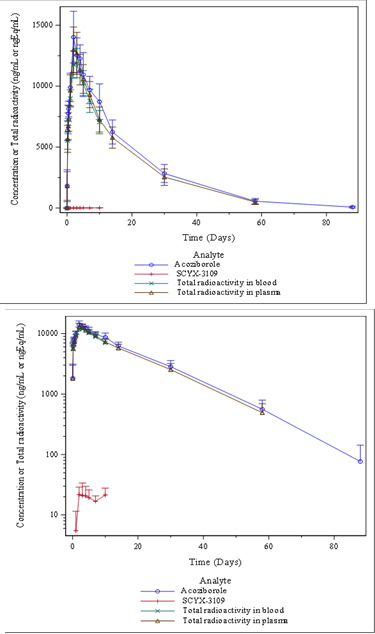
Mean concentration-time profiles of total radioactivity in blood and of acoziborole, SCYX-3109, and total radioactivity in plasma over time (*N* = 6; upper panel: linear/linear and lower panel: logarithmic/linear representations).

Extraction efficiency for plasma was 98.3%, indicating no noticeable irreversible binding to or sequestration by plasma components. Mean concentration-time profiles of total radioactivity in blood and plasma, and of acoziborole and its metabolite SCYX-3109 in plasma, are presented in Fig. 2. A summary of selected PK parameters is reported in Table 2.

**TABLE 2:** Non-compartmental pharmacokinetics of total radioactivity, acoziborole, and its metabolite SCYX-3109 in plasma and whole blood (*N* = 6)^*a*^^,^^*b*^

Parameter	Total radioactivity		Acoziborole in plasma	SCYX-3109 in plasma
	Whole blood	Plasma		
*C*_max_ (µg/mL or µg-eq/mL)	12.052 ± 1.129	13.130 ± 1.776	14.200 ± 1.980	0.0305 ± 0.0107
*t*_max_ (h)	68.1 ± 9.9	56.0 ± 12.4	52.0 ± 9.8	76.1 ± 28.0
AUC_0–240h_ (µg*h/mL or µg-eq*h/mL)	2,250.737 ± 182.816	2,371.786 ± 255.572	2,519.779 ± 255.918	4.373 ± 1.025
AUC_0–*t*_ (µg*h/mL or µg-eq*h/mL)	3,556.202 ± 3,236.208	5,403.286 ± 1,561.755	5,920.945 ± 1,562.723	3.732 ± 2.324
AUC_0–∞_ (µg*h/mL or µg-eq*h/mL)	5,382.336 ± 2,473.408	5,785.324 ± 1,105.726	6,450.774 ± 1,044.913	NC
*t*_1/2_ (h)	239.2 ± 65.7	278.3 ± 47.4	272.1 ± 36.3	NC
CL/F (L/h)	NC	NC	0.2 ± 0.0	NC
*V*_z_/*F* (L)	NC	NC	59.1 ± 7.3	NC

^*a*^ Arithmetic means and standard deviations are shown. *C*_max_ is presented in μg/mL for acoziborole and SCYX-3109, and in μg-eq/mL for total radioactivity. AUC_0–240h_, AUC_0–*t*_and AUC_0-∞ _are presented in μg*h/mL for acoziborole and SCYX-3109, and in μg-eq*h/mL for total radioactivity.

^*b*^ AUC: area under the plasma concentration-time curve, AUC_0-240h_: AUC from time 0 (pre-dose) to 240 h post-dose, AUC_0–*t*_: AUC from time 0 to the time of the last measurable concentration, AUC_0–∞_: AUC from time 0 to infinity, *C*_max_: maximum concentration, CL/F: apparent total body clearance, NA: not available, NC: not calculable, *t*_1/2_: elimination half-life, *t*_max_: time to reach *C*_max_, *V*_z_/*F:* apparent volume of distribution during the terminal phase.

Total radioactivity in blood and plasma, and acoziborole in plasma, were measurable in the first sample collected one hour after dosing in all participants. The median *t*_max_ was 48 h for both acoziborole and total radioactivity in plasma, with individual values between 48 and 72 h.

The concentration-time profiles of acoziborole and total radioactivity in plasma were comparable from 0 to 1,440 h (Day 61). However, as study participants remained in the clinic unit up to 240 h after acoziborole administration, PK parameters were compared during that time period mainly. *C*_max_ and AUC_0–240h_ values for acoziborole and total radioactivity in plasma were similar (*C*_max_: 14.20 µg/mL and 13.13 µg-eq/mL; AUC_0–240h_: 2,519.78 µg*h/mL and 2,250.74 µg-eq*h/mL, respectively). Consequently, based on the AUC_0–240h_ and AUC_0–∞_ ratios of acoziborole to total radioactivity in plasma, acoziborole accounted for almost 100% of the radioactivity in plasma. SCYX-3109 was first detected in plasma between 24 and 48 h after [^14^C]-acoziborole administration and was quantifiable in all participants between 48 and 336 h post-dose. Over the 240-h period, mean concentrations of SCYX-3109 in plasma were minimal, representing 0.2% of acoziborole plasma levels based on the AUC_0–240h_ ratio.

Mean total radioactivity revealed qualitatively similar concentration-time profiles in blood and plasma (Fig. 2), with mean concentrations of total radioactivity in whole blood comparable to those in plasma. Estimated terminal *t*_1/2_ values (Table 2) of acoziborole in plasma and total radioactivity in plasma and blood were also similar, with mean values of 272.1, 278.3, and 239.2 h, respectively, resulting in mean *t*_1/2_ ratios of acoziborole to total radioactivity in plasma and blood of 1.0 and 1.1, respectively.

Total radioactivity in plasma was still quantifiable above the LLOQ in two out of five participants on Day 89; however, none of the participants had quantifiable total radioactivity in plasma on the last sample day (Day 119).

### Excretion and recovery of radioactivity

**Fig 3:**
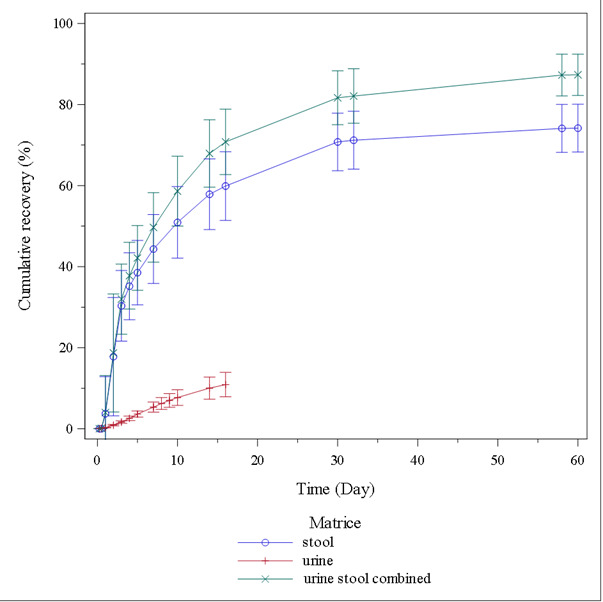
Mean percentage cumulative urine, feces (stool), and total recovery over time for unchanged acoziborole-related total radioactivity (*N* = 6).

**Fig 4:**
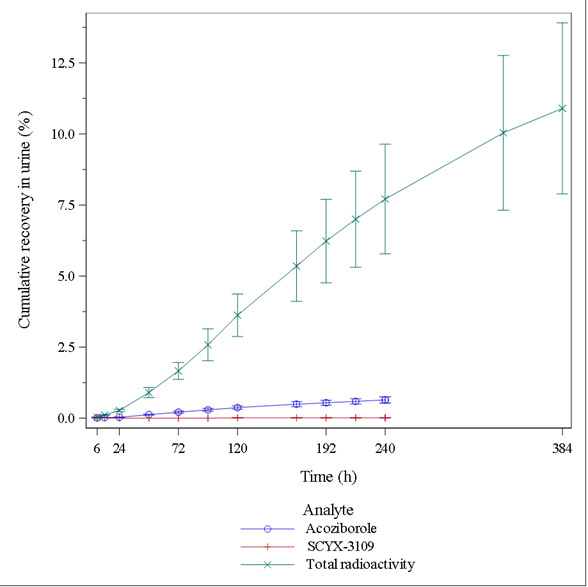
Mean percentage cumulative recovery in urine over time for unchanged acoziborole, SCYX-3109, and acoziborole-related total radioactivity (*N* = 6).

For fecal homogenates and urine (centrifugation only), extraction efficiency was 76.0% and 108.7%, respectively. Total radioactivity excreted and mass balance are presented in the Supplementary Information section 4, and cumulative proportions of radioactivity recovered from feces (up to 1,440 h) and from urine (up to 384 h) are displayed in Fig. 3. After oral administration of 960 mg [^14^C]-acoziborole, the recovered radioactivity was mainly detected in feces. The mean Cum%Ae (±SD) of total radioactivity in feces was 50.9 ± 8.8%, 59.9 ± 8.5%, and 74.2 ± 5.9% of the total dose after 240, 384, and 1,440 h, respectively. The mean Cum%Ae (±SD) of total radioactivity in urine was 7.7 ± 1.9% and 10.9 ± 3.0% of the total dose after 240 and 384 h, respectively. Over the total 1,392 h, the geometric mean recovery of total radioactivity in urine and feces was 87.2%, with low CV% (5.5%) and less than a 1% increment in excretion up to 1,440 h. Mean percentage cumulative recovery in urine over time is presented in Fig. 4. Acoziborole and SCYX-3109 renal clearances (CLr_0–240h_ ± SD) were 0.002 ± 0.000 and 0.036 ± 0.021 L/h, respectively.

### Metabolite profiling and identification

**Fig 5:**
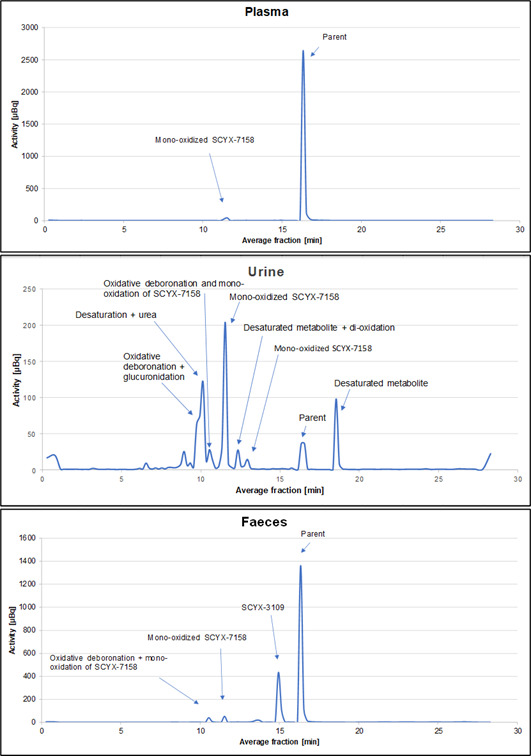
Representative radioactivity chromatograms per fraction (represented as µBq) showing metabolic profiles for 0–336 h plasma pool, 0–240 h urine pool, and 0–240 h fecal homogenate pool.

**Fig 6:**
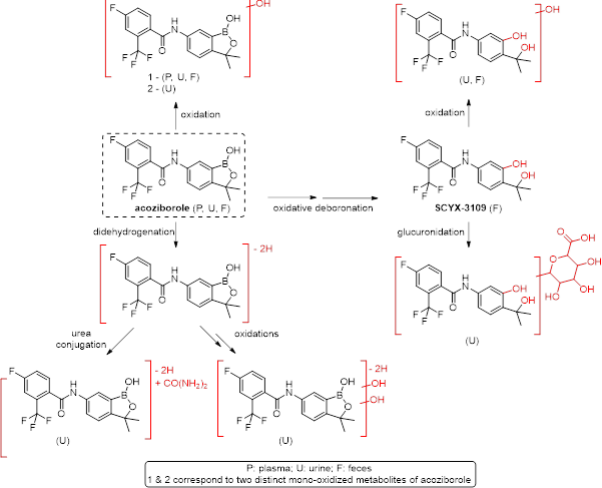
Putative metabolic scheme for [^14^C]-acoziborole following a single oral dose of 960 mg [^14^C]-acoziborole [37 kBq] to healthy participants.

Radiochromatograms of pooled plasma, urine, and fecal homogenate samples are shown in Fig. 5. Corresponding relative abundance of radiochromatogram peaks in these matrices, as well as abundance relative to dose for urine and feces, is presented in Table 3. Acoziborole and its metabolite SCYX-3109 were identified based on the retention time of the reference standards. Other identities were tentatively assigned based on the observed and calculated mass. A proposed metabolite scheme for [^14^C]-acoziborole following administration of a single oral dose of 960 mg of [^14^C]-acoziborole is presented in Fig. 6.

**TABLE 3:** Relative abundances of radiochromatogram peaks in pools of plasma, urine, and feces

Observed retention time [min]	Proposed identity	Plasma^*a*^ (0–336 h)	Urine^*a*^ (0–240 h)	Feces homogenates^*a*^ (0–240 h)
6.5	Unknown	–^*c*^	1.1% (0.1%)	–
8.9	Unknown	–	4.0% (0.3%)	–
9.1	Unknown	–	1.8% (0.1%)	–
9.9	Oxidative deboronation + glucuronidation	–	27.7% (2.1%)	–
10.2	Desaturation + urea			
10.7	Oxidative deboronation + mono-oxidation of acoziborole	–	5.6% (0.4%)	2.2% (1.1%)
11.6	Mono-oxidized acoziborole	2.3%	27.2% (2.1%)	2.4% (1.2%)
12.5	Desaturated metabolite + di-oxidation of acoziborole	–	3.5% (0.3%)	–
12.9	Mono-oxidized acoziborole	–	2.2% (0.2%)	–
15.1	SCYX-3109	–	–	24.2% (12.3%)
16.5	Parent (acoziborole)	95.1%	7.6% (0.6%)	66.0% (33.6%)
18.6	Desaturated metabolite	–	11.3% (0.9%)	–
Total assigned		97.3%	92.0% (7.1%)	94.8% (48.3%)
Unassigned^*b*^		2.7%	8.0% (0.6%)	5.2% (2.6%)
Total		100%	100% (7.7%)	100% (50.9%)

^*a*^ Values indicate relative abundance radiochromatogram for plasma, or relative abundance radiochromatogram and in parentheses abundance relative to dose for urine and feces homogenates. Abundance relative to dose was calculated using the following equation: Abundance relative to dose = relative abundance (%)/cumulative dose excreted in matrix (%) × 100%.

^*b*^ Sum of radioactivity detected above background in all fractions.

^*c*^ "–”, indicates that no data is available.

### Plasma

Acoziborole accounted for most of the radioactivity in plasma (95.1%; Fig. 5). Only one other peak, accounting for 2.3% of the radioactivity, was observed and assigned as mono-oxidized acoziborole, based on the observed molecular weight of the corresponding metabolite. Unassigned peaks (sum of radioactivity detected above background) accounted for 2.7% of the radioactivity.

### Urine

Acoziborole and seven metabolites were detected in urine (Fig. 5). Acoziborole and mono-oxidized acoziborole represented 0.6% and 2.3% of the dose, respectively. The five other identified metabolites (including oxidative deboronation + glucuronidation and desaturated metabolites) represented 3.7% of the dose in total.

### Feces

Acoziborole was the most abundant entity in feces, representing 33.6% of the dose (Fig. 5). Three additional peaks were observed: SCYX-3109 was the most abundant, representing 12.3% of the dose, while the remaining were tentatively assigned as oxidized, deboronated, and mono-oxidized acoziborole, altogether representing 2.3% of the dose.

Overall, the sum of total radioactivity detected above background in all fractions, which was not tentatively assigned to any entity, was 8.0% (abundance relative to dose of 0.6%) in urine and 5.2% (abundance relative to dose of 2.6%) in feces (Table 3).

## DISCUSSION

This study aimed to determine (i) the excretion balance and systemic exposure of total circulating radioactivity in blood and plasma after oral administration of [^14^C]-acoziborole, (ii) the PK of acoziborole and SCYX-3109 in plasma by LC-MS/MS for comparison with total circulating radioactivity, and (iii) the metabolic pathways of acoziborole in plasma, urine, and feces.

The changes in the conduct of the study due to the global COVID-19 pandemic and restrictions on travels did not compromise the study’s main objectives, even if they may have contributed to slightly underestimating the recovery of the radioactivity in urine.

The recovery of radioactivity in excreta over 60 days was satisfactory, 87.3% on average. Excretion via feces accounted for the major elimination pathway of the administered radioactive dose. On average, 74.2% of the dose was excreted in feces and 10.9% was excreted in urine.

In an *in vitro* MDCK-MDR1 cell monolayer assay, acoziborole showed a high absorption potential (with an apparent permeability of 776 nm/s) (1). These data suggest that in humans, intestinal permeability may not be a limiting factor for acoziborole absorption. In this study in healthy participants, acoziborole absorption began rapidly after oral administration, as evidenced by radioactivity (in blood and plasma) and acoziborole (in plasma) being detected 1 h after dosing. However, *C*_max_ was reached after 48 h (median *t*_max_) only, in line with what was reported elsewhere (8). An intravenous formulation of acoziborole was not available, and absolute acoziborole bioavailability could not be determined. The absorbed fraction in fasting condition was therefore estimated (assuming a mean standard gastrointestinal transit time of approximately 3 days up to feces) to be 70% of the dose or more, since most of the radioactivity was excreted in feces more than 48 hours after [^14^C]-acoziborole administration. This estimate is broadly in line with what was reported in NHPs (89%) (1) and with the results of a physiologically based PK modeling study that, using the fully mechanistic absorption model in GastroPlus (ACAT), estimated the fraction absorbed ranging between 74% (CV%: 14%) and 79% (CV%: 18%) in healthy subjects and patients with g-HAT.

Acoziborole was the main component of the radioactivity in plasma, as concentration-time profiles of both acoziborole and radioactivity up to 1,440 h were almost superimposable. In addition, the mean whole blood to plasma total radioactivity ratios (for *C*_max_ and AUC_0–240h_) suggested that acoziborole has no affinity for blood cells. The volume of distribution *V*_z_/*F* (59.1 ± 7.3 L) suggested compound distribution beyond the central compartment.

In a pool of plasma samples collected over 14 days, acoziborole accounted for 95.1% of total radioactivity, while only a mono-oxidized form of acoziborole was identified, accounting for 2.3%, suggesting that metabolism of [^14^C]-acoziborole is limited and slow. In particular, SCYX-3109 could not be detected. As presented in Fig. 6, the major pathways of acoziborole metabolism identified in humans include oxidative deboronation of acoziborole (SCYX-3109, 12.3%) with further glucuronidation (2.1%) or mono-oxidation (1.5%) and mono-oxidation of acoziborole (3.5%). Acoziborole predominantly underwent slow liver/biliary-fecal elimination, and 66.0% of the excreted radioactivity (33.6% relative to the dose) was detected in feces over 10 days, while its metabolite SCYX-3109 accounted for 24.2% (12.3% of the dose). Of note, proportions relative to dose were probably underestimated as excretion of radioactivity was not complete after 10 days. The absence of profiling in later samples was, however, not of concern, as the half-life of the total radioactivity (278 h) was similar to that of acoziborole (272 h), meaning that no metabolites had a slower elimination rate limited clearance. In addition, since acoziborole accounted for more than 95% of the total radioactivity in plasma, no further metabolite profiling and investigations were deemed necessary.

Overall, acoziborole appeared to be slowly eliminated, mainly by the biliary-fecal route with low metabolism. Its renal excretion was minor, with numerous metabolites accounting for the radioactivity excreted in urine.

Acoziborole was shown to be metabolically stable when incubated *in vitro* with liver sub-cellular fractions from rodents, dogs, cynomolgus monkeys, and humans, and *in vitro* metabolism of acoziborole was shown to be limited over time across these species (1). The main metabolites of acoziborole detected in human primary hepatocytes were SCYX-3109 with further glucurono-conjugation (1). These metabolites were also detected in rat and dog hepatocytes. After [^14^C]-acoziborole administration (at 10 mg/kg [200 mCi/kg] dose level) to Sprague-Dawley rats, five metabolites were detected by LC-MS/MS, including mono-oxidized acoziborole, SCYX-3109, mono-oxidized and di-oxidized SCYX-3109, and glucuronidation products of SCYX-3109.

Overall, the metabolism of acoziborole appeared very limited in this study in healthy participants. The major pathways of acoziborole metabolism identified in humans were also observed *in vivo* in rats.

Most oxidative transformations of drugs and xenobiotics are catalyzed by CYP450 enzymes (12, 13), including oxidative deboronation as described with, for example, the proteasome inhibitor bortezomib (14). In line with the above, *in vitro* incubation of 1 µM acoziborole with CYP450 recombinant enzymes (Bactosomes) expressing human CYPs and nicotinamide adenine dinucleotide phosphate oxidase (NADPH) showed that acoziborole was metabolized by CYP1A2, CYP2C8, and CYP3A4 and not by CYP2B6, CYP2C9, CYP2C19, CYP2D6, and CYP3A5 recombinant enzymes.

Comparative studies of African populations have revealed great genetic heterogeneity of CYP450 genes (15) and allele frequency differences, notably in comparison with Europeans and Asians (16). This genetic structuring may be associated with differences in the success of drug therapies (15). However, evidence regarding the genetic variability of CYP1A2, CYP2C8, and CYP3A4 and their clinical implications is not as conclusive as for other CYPs. Overall, such genetic heterogeneity appears to be a minor concern for acoziborole, given its limited oxidative metabolism. The acoziborole metabolite profile reported in Caucasian healthy subjects is not expected to be different in the African population.

In conclusion, the present study showed that acoziborole is weakly and slowly metabolized in humans, and it was the main circulating compound in plasma. Overall, 34.2% of the dose was excreted as unchanged acoziborole, mainly in feces (33.6%), whereas about 20.6% of the dose was excreted as identified metabolites and 3.7% as unassigned or unknown. Acoziborole had a predominant but slow biliary-fecal elimination with limited metabolism and a very low renal elimination (<1% of the dose as unchanged drug).

## Data Availability

The data underlying the results of this study are available upon request because they contain potentially sensitive personal information, which must be de-identified at the individual level. Interested researchers may request access to de-identified participant data from Vivli, the data-sharing partner of the Drugs for Neglected Diseases initiative (DNDi), commissioner of this study, at https://vivli.org/ourmember/dndi/.

## SUPPLEMENTAL MATERIAL

Supplemental material aac.00580-25-s0001.docx Inclusion and exclusion criteria, clinical laboratory parameters, incidence of treatment-emergent adverse events, total radioactivity excreted, and mass balance.

ASM does not own the copyrights to Supplemental Material that may be linked to, or accessed through, an article. The authors have granted ASM a non-exclusive, world-wide license to publish the Supplemental Material files. Please contact the corresponding author directly for reuse.

## References

[1] Jacobs RT, Nare B, Wring SA, Orr MD, Chen D, Sligar JM, Jenks MX, Noe RA, Bowling TS, Mercer LT, et al.. 2011. SCYX-7158, an orally-active benzoxaborole for the treatment of stage 2 human African trypanosomiasis. PLoS Negl Trop Dis 5:e1151. doi:10.1371/journal.pntd.0001151 21738803 PMC3125149

[2] Brun R, Don R, Jacobs RT, Wang MZ, Barrett MP. 2011. Development of novel drugs for human African trypanosomiasis. Future Microbiol 6:677–691. doi:10.2217/fmb.11.44 21707314

[3] Brun R, Blum J, Chappuis F, Burri C. 2010. Human African trypanosomiasis. Lancet 375:148–159. doi:10.1016/S0140-6736(09)60829-1 19833383

[4] Kennedy PGE. 2019. Update on human African trypanosomiasis (sleeping sickness). J Neurol 266:2334–2337. doi:10.1007/s00415-019-09425-7 31209574

[5] Kennedy PGE, Rodgers J. 2019. Clinical and neuropathogenetic aspects of human African trypanosomiasis. Front Immunol 10:39. doi:10.3389/fimmu.2019.00039 30740102 PMC6355679

[6] Wall RJ, Rico E, Lukac I, Zuccotto F, Elg S, Gilbert IH, Freund Y, Alley MRK, Field MC, Wyllie S, Horn D. 2018. Clinical and veterinary trypanocidal benzoxaboroles target CPSF3. Proc Natl Acad Sci USA 115:9616–9621. doi:10.1073/pnas.1807915115 30185555 PMC6156652

[7] Tarral A, Hovsepian L, Duvauchelle T, Donazzolo Y, Latreille M, Felices M, Gualano V, Delhomme S, Valverde Mordt O, Blesson S, Voiriot P, Strub-Wourgaft N. 2023. Determination of the optimal single dose treatment for acoziborole, a novel drug for the treatment of human African trypanosomiasis: first-in-human study. Clin Pharmacokinet 62:481–491. doi:10.1007/s40262-023-01216-8 36763327 PMC10042906

[8] Betu Kumeso VK, Kalonji WM, Rembry S, Valverde Mordt O, Ngolo Tete D, Prêtre A, Delhomme S, Ilunga Wa Kyhi M, Camara M, Catusse J, et al.. 2023. Efficacy and safety of acoziborole in patients with human African trypanosomiasis caused by Trypanosoma brucei gambiense: a multicentre, open-label, single-arm, phase 2/3 trial. Lancet Infect Dis 23:463–470. doi:10.1016/S1473-3099(22)00660-0 36460027 PMC10033454

[9] International Atomic Energy Agency. 2014. International Atomic Energy Agency, Reference Sheet for IAEA-C1 to IAEA-C9 Quality Control Materials. IAEA, Vienna. Available from: https://nucleus.iaea.org/sites/ReferenceMaterials/Shared%20Documents/ReferenceMaterials/Radionuclides/IAEA-C-1/RS_IAEA-C1_to_IAEA-C9_Rev_01.pdf. Retrieved 20 Oct 2023.

[10] Administration of Radioactive Substances Advisory Committee. 2023. Notes for guidance on the clinical administration of radiopharmaceuticals and use of sealed radioactive sources. UKHSA publications gateway number GOV-14149. Available from: https://www.gov.uk/government/publications/arsac-notes-for-guidance. Retrieved 20 Oct 2023.

[11] Dickie EA, Giordani F, Gould MK, Mäser P, Burri C, Mottram JC, Rao SPS, Barrett MP. 2020. New drugs for human African trypanosomiasis: a twenty first century success story. Trop Med Infect Dis 5:29. doi:10.3390/tropicalmed5010029 32092897 PMC7157223

[12] Rendic S, Guengerich FP. 2015. Survey of human oxidoreductases and cytochrome P450 enzymes involved in the metabolism of xenobiotic and natural chemicals. Chem Res Toxicol 28:38–42. doi:10.1021/tx500444e 25485457 PMC4303333

[13] Guengerich FP. 2018. Mechanisms of cytochrome P450-catalyzed oxidations. ACS Catal 8:10964–10976. doi:10.1021/acscatal.8b03401 31105987 PMC6519473

[14] Labutti J, Parsons I, Huang R, Miwa G, Gan LS, Daniels JS. 2006. Oxidative deboronation of the peptide boronic acid proteasome inhibitor bortezomib: contributions from reactive oxygen species in this novel cytochrome P450 reaction. Chem Res Toxicol 19:539–546. doi:10.1021/tx050313d 16608165

[15] Bains RK. 2013. African variation at Cytochrome P450 genes: evolutionary aspects and the implications for the treatment of infectious diseases. Evol Med Public Health 2013:118–134. doi:10.1093/emph/eot010 24481193 PMC3868406

[16] Dandara C, Lombard Z, Du Plooy I, McLellan T, Norris SA, Ramsay M. 2011. Genetic variants in CYP (-1A2, -2C9, -2C19, -3A4 and -3A5), VKORC1 and ABCB1 genes in a black South African population: a window into diversity. Pharmacogenomics 12:1663–1670. doi:10.2217/pgs.11.106 22118051

