# Supplementary material for: Mass balance, pharmacokinetics, metabolism, and excretion of radiolabeled acoziborole, a potential novel treatment for human African trypanosomiasis, following single microtracer oral dose to humans
Source: Antimicrob Agents Chemother. 2025 Sep 22;69(11):e00580-25. doi: 10.1128/aac.00580-25 (PMC12587574; doi:10.1128/aac.00580-25)
Supplement: Supplemental material — Inclusion and exclusion criteria, clinical laboratory parameters, incidence of treatment-emergent adverse events, total radioactivity excreted, and mass balance. [file aac.00580-25-s0001.docx]

**SUPPLEMENTARY INFORMATION**

**Inclusion and exclusion criteria**

**Inclusion criteria**

1. Healthy white males.

2. Age 18 to 55 years of age at the time of signing informed consent.

3. Body mass index (BMI) of 18.0 to 30.0 kg/m^2^ as measured at screening.

4. Must be willing and able to communicate and participate in the whole study.

5. Must have regular bowel movements (i.e., average stool production of ≥1 and ≤3 stools per day).

6. Normal blood pressure (BP): systolic BP between 90 and 140 (160 if >45 years old) mmHg (inclusive), diastolic BP 45 to 90 mmHg (inclusive), measured after 10 min rest in supine position at screening and pre-dose.

7. A resting heart rate between 45 and 100 bpm (inclusive), measured after 10 min rest in supine position at screening and pre-dose.

8. Electrocardiogram recording without clinically significant abnormality, including QT intervals corrected with Fridericia’s formula (QTcF) measure of ≤450 msec at screening and pre-dose.

9. Must provide written informed consent.

10. Must agree to adhere to the contraception requirements.

11. Must be able to swallow multiple capsules.

Inclusion criteria 5, 6, 7, 8 and 10 from the list above were re-assessed at admission/pre-dose.

**Exclusion Criteria**

1. Participants who had received any investigational medicinal product (IP) in a clinical research study within the 90 days prior to Day 1.

2. Participants who were study site employees, or immediate family members of a study site or sponsor employee.

3. History of any drug or alcohol abuse in the past 2 years.

4. Regular alcohol consumption >21 units per week and (1 unit = ½ pint beer, or a 25 mL shot of 40% spirit, 1.5 to 2 units = 125 mL glass of wine, depending on type) as confirmed by a positive alcohol breath test at screening or any on admission to the clinical unit.

5. Current smokers and those who had smoked within the last 6 months. As confirmed by a breath carbon monoxide reading of greater than 10 ppm at screening or admission.

6. Participants with pregnant or lactating partners.

7. Radiation exposure, including that from the present study, excluding background radiation but including diagnostic X-rays and other medical exposures, exceeding 5 mSv in the last 12 months or 10 mSv in the last 5 years. No occupationally exposed worker, as defined in [confidential unpublished information], could participate in the study.

8. Participants who had been enrolled in an absorption, distribution, metabolism and excretion (ADME) study in the last 12 months.

9. Participants who did not have suitable veins for multiple venepunctures/cannulation as assessed by the investigator or delegate at screening.

10. Clinically significant abnormal clinical chemistry, haematology, urinalysis (especially alanine aminotransferase, aspartate aminotransferase and alkaline phosphatase) or clinically significant abnormal physical examination findings as judged by the investigator.

11. Abnormal thyroid function test results.

12. Abnormal renal function (estimate glomerular filtration rate [eGFR] <80 mL/min).

13. Confirmed positive drugs of abuse test result.

14. Positive hepatitis B surface antigen, hepatitis C virus antibody or human immunodeficiency virus (HIV) results.

15. History of any clinically significant acute or chronic cardiovascular, renal, hepatic, neurological (especially seizures), immunological, psychiatric, myopathies, bleeding tendency, respiratory and particularly gastrointestinal (GI) disease, especially peptic ulceration and chronic gastritis, GI bleeding, ulcerative colitis, Crohn’s disease or irritable bowel syndrome, as judged by the investigator.

16. Any relevant GI complaints within 7 days of dosing.

17. Serious adverse reaction or serious hypersensitivity to any drug or the formulation excipients.

18. Presence or history of clinically significant allergy requiring treatment (including asthma, urticaria, clinically significant allergic rash or other severe allergic diathesis), as judged by the investigator. Hay fever was allowed unless it was active.

19. Donation or loss of greater than 400 mL of blood within the previous 3 months or more than 100 mL within 30 days before signing informed consent to this trial.

20. Participants who were taking any prescribed drug in the 14 days before screening or require regular use of any prescription medication during the study.

21. Participants who had taken any over-the-counter medications, including vitamins, analgesics or antacids, herbal remedies or St. John’s wort in the 7 days before IP administration. Exceptions may apply on a case-by-case basis, if considered not to interfere with the objectives of the study, as determined by the principal investigator.

22. Use of enzyme-altering drugs (e.g. barbiturates, phenothiazines, cimetidine) within 30 days or 5 half-lives, whichever was longer, of study Day 1.

23. Surgery within 12 weeks prior to screening, with the exception of appendectomy or at the discretion of the investigator for minor surgery.

24. Any surgery (e.g. gastric bypass) or medical condition that may affect absorption of orally administered drugs.

25. Failure to satisfy the investigator of fitness to participate for any other reason.

Exclusion criteria 4, 5, 13, 15, 16, 20, 21, 22 and 25 from the list above were re-assessed at admission/pre-dose.

**Clinical laboratory parameters**

| **Haematology** | **Clinical Chemistry** | **Virology** | **Urinalysis** | **Drugs of Abuse** |
| --- | --- | --- | --- | --- |
| Basophils  Eosinophils  Haematocrit  (packed cell volume- PCV)  Haemoglobin  Lymphocytes  Mean corpuscular haemoglobin (MCH)  Mean corpuscular haemoglobin concentration (MCHC)  Mean corpuscular volume (MCV)  Monocytes  Neutrophils  Platelet count  Red blood cell (RBC) count  White blood cell (WBC) | Alanine aminotransferase (ALT)  Albumin  Alkaline phosphatase  Aspartate aminotransferase (AST)  Bicarbonate  Bilirubin (total)  Bilirubin (direct) (only if total is elevated)  Calcium  Chloride  Creatine kinase (CK)  Creatinine  Gamma glutamyl transferase (GGT)  Glucose  Glucose (fasting)  Phosphate (inorganic)  Potassium  Protein (total)  Sodium  Urea  **Thyroid Function Tests**  Thyroid-stimulating hormone (TSH)  Free triiodothyronine (FT3)  Free thyroxine (FT4) | Hepatitis B surface antigen  Hepatitis C antibody  HIV 1 & 2 antibodies | Bilirubin  Blood  Glucose  Ketones  Leukocytes  Nitrites  pH  Protein  Specific gravity  Urobilinogen  **At discretion of investigator based on urinalysis results**  Microbiology  Urine microscopy | Amphetamines  Barbiturates  Benzodiazepines  Cocaine  Marijuana/cannabis  Methadone  Methamphetamine/ecstasy  Morphine/opiates  Phencyclidine  Tricyclic antidepressants |

**Incidence of treatment-emergent adverse events by system organ class and time of occurrence**

| System organ class | Day 1 | | Day 2 to Day 15 | | Day 16 to end of study | |
| --- | --- | --- | --- | --- | --- | --- |
| Preferred term | n (%) | Events | n (%) | Events | n (%) | Events |
| TEAEs | 2 (33.3) | 2 | 4 (66.7) | 6 | 3 (50.0) | 5 |
| Infections and infestation | 0 (0.0) | 0 | 1 (16.7) | 2 | 1 (16.7) | 1 |
| Balanitis candida  Rhinitis | 0 (0.0)  0 (0.0) | 0  0 | 1 (16.7)  0 (0.0) | 2  0 | 0 (0.0)  1 (16.7) | 0  1 |
| Investigations | 0 (0.0) | 0 | 1 (16.7) | 1 | 1 (16.7) | 1 |
| Phosphokinase increased  Blood glucose increased | 0 (0.0)  0 (0.0) | 0  0 | 1 (16.7)  0 (0.0) | 1  0 | 0 (0.0)  1 (16.7) | 0  1 |
| Nervous system disorders | 2 (33.3) | 2 | 1 (16.7) | 1 | 1 (16.7) | 1 |
| Headache | 2 (33.3) | 2 | 1 (16.7) | 1 | 1 (16.7) | 1 |
| Gastrointestinal disorders | 0 (0.0) | 0 | 1 (16.7) | 1 | 0 (0.0) | 0 |
| Toothache | 0 (0.0) | 0 | 1 (16.7) | 1 | 0 (0.0) | 0 |
| Injury, poisoning and procedural complications | 0 (0.0) | 0 | 0 (0.0) | 0 | 1 (16.7) | 1 |
| Skin laceration | 0 (0.0) | 0 | 0 (0.0) | 0 | 1 (16.7) | 1 |
| Musculoskeletal and connective tissue disorders | 0 (0.0) | 0 | 0 (0.0) | 0 | 1 (16.7) | 1 |
| Myalgia | 0 (0.0) | 0 | 0 (0.0) | 0 | 1 (16.7) | 1 |
| Surgical and medical procedures | 0 (0.0) | 0 | 1 (16.7) | 1 | 0 (0.0) | 0 |
| Tooth repair | 0 (0.0) | 0 | 1 (16.7) | 1 | 0 (0.0) | 0 |

TEAEs: treatment-emergent adverse events

N = 6 participants

**Total radioactivity excreted and mass balance (N = 6)**

| **PK parameter** | **Total radioactivity** | | |
| --- | --- | --- | --- |
|  | **In faeces** | **In urine** | **Recovered** |
| CumAe_0-240h_ (mgEq) | 488.8 + 84.8 | 74.0 + 18.5 | 562.9 + 82.8 |
| Cum%Ae_0-240h_ dose (%) | 50.9 + 8.8 | 7.7 + 1.9 | 58.6 + 8.6 |
| CumAe_0-384h_ (mgEq) | 574.9 + 81.4 | 104.6 + 28.9 | 679.6 + 77.5 |
| Cum%Ae_0-384h_ dose (%) | 59.9 + 8.5 | 10.9 + 3.0 | 70.8 + 8.1 |
| CumAe_0-t_ (mgEq)^a^ | 712.1 + 56.4 | 104.6 + 28.9 | 838.4 + 48.9 |
| Cum%Ae_0-t_ dose^a^ (%) | 74.2 + 5.9 | 10.9 + 3.0 | 87.3 + 5.1 |

Arithmetic means and standard deviations are shown.

^a^Due to sanitary restrictions related to the COVID-19 pandemic, collection of urine had to be limited to 384 h, while faeces were collected up to 2880 h. Only 2 participants had radioactivity quantifiable in stools after 1440 h (60 days) and *t* was set to 1440 h.

*CumAe* cumulative amount excreted, *Cum%Ae dose* recovered cumulative percentage of dose, *t* time of last measurable post-dose concentration.
